# Resveratrol suppresses NTHi-induced inflammation via up-regulation of the negative regulator MyD88 short

**DOI:** 10.1038/srep34445

**Published:** 2016-09-28

**Authors:** Carla S. Andrews, Shingo Matsuyama, Byung-Cheol Lee, Jian-Dong Li

**Affiliations:** 1 Center for Inflammation, Immunity & Infection, Institute for Biomedical Sciences, Georgia State University, Atlanta, GA, 30303, USA

## Abstract

Upper respiratory tract inflammatory diseases such as asthma and chronic obstructive pulmonary diseases (COPD) affect more than one-half billion people globally and are characterized by chronic inflammation that is often exacerbated by respiratory pathogens such as nontypeable *Haemophilus influenzae* (NTHi). The increasing numbers of antibiotic-resistant bacterial strains and the limited success of currently available pharmaceuticals used to manage the symptoms of these diseases present an urgent need for the development of novel anti-inflammatory therapeutic agents. Resveratrol has long been thought as an interesting therapeutic agent for various diseases including inflammatory diseases. However, the molecular mechanisms underlying its anti-inflammatory properties remain largely unknown. Here we show for the first time that resveratrol decreases expression of pro-inflammatory mediators in airway epithelial cells and in the lung of mice by enhancing NTHi-induced MyD88 short, a negative regulator of inflammation, via inhibition of ERK1/2 activation. Furthermore, resveratrol inhibits NTHi-induced ERK1/2 phosphorylation by increasing MKP-1 expression via a cAMP-PKA-dependent signaling pathway. Finally, we show that resveratrol has anti-inflammatory effects post NTHi infection, thereby demonstrating its therapeutic potential. Together these data reveal a novel mechanism by which resveratrol alleviates NTHi-induced inflammation in airway disease by up-regulating the negative regulator of inflammation MyD88s.

Upper respiratory tract inflammatory diseases such as asthma and chronic obstructive pulmonary diseases (COPD) affect more than one-half billion people globally. Asthma is reported to result in 250 thousand deaths annually and is the leading cause of hospitalizations among children less than 15 years of age in the U.S.^1^,^2^,^3^. COPD is currently the third leading cause of death in the U.S. The World Health Organization predicts that by the year 2020, COPD will be the fifth most significant contributor to the worldwide burden of disease^4^. Billions of dollars in healthcare costs are associated with these diseases and there are no cures^1^,^5^. These airway diseases are characterized by chronic inflammation resulting in epithelial injury and airflow limitation and exacerbations are often caused by respiratory pathogens such as nontypeable *Haemophilus influenzae* (NTHi)^6^,^7^. Antibiotics are routinely used to treat NTHi infections. However, the increasing numbers of antibiotic-resistant strains present an urgent need for the development of non-antibiotic therapeutics^8^,^9^,^10^,^11^. Common pharmaceuticals used to manage the symptoms of asthma and COPD include β2-agonists and inhaled corticosteroids. Success of these therapeutics and others is limited due to adverse side effects, tolerance and cost as evidenced by the increasing prevalence of disease^1^,^4^. Therefore, novel therapeutics are urgently needed.

Airway epithelial cells are ultimately responsible for the major symptomatic pathology, namely inflammation, associated with asthma and COPD^12^,^13^. During disease exacerbations, epithelial cells recognize and respond to antigens via pattern recognition receptors (PRRs) called toll-like receptors (TLRs). NTHi binds to toll-like receptor 2 (TLR2) on epithelial cells initiating a signaling cascade resulting in the production of antimicrobial peptides as well as cytokines and chemokines^14^,^15^,^16^,^17^. Following TLR2 activation by NTHi recognition, the adaptor protein myeloid differentiation factor 88 (MyD88) is recruited to the receptor. MyD88 recruitment leads to activation of nuclear factor-kappa B (NF-κB) and mitogen-activated protein kinases (MAPKs) to enhance the inflammatory response^18^. MyD88 is recruited to many TLRs and is critical for host defense to a variety of pathogen-associated molecular patterns (PAMPs). Therefore, immune homeostasis requires tight control of MyD88-mediated signaling pathways.

An alternatively spliced variant of MyD88, called MyD88 short (MyD88s), has been identified to be a negative regulator of inflammatory signaling pathways. MyD88s cannot recruit and phosphorylate IL-1 receptor-associated kinases (IRAKs) and therefore cannot activate MAPK and NF-κB^19^,^20^,^21^. We have previously shown that NTHi induces MyD88s expression and MyD88s is a negative regulator of inflammation in airway epithelial cells. We further demonstrated that MyD88s is positively regulated by I kappa B kinase beta (IKKβ) and the cyclic AMP (cAMP) response element-binding protein (CREB) and negatively regulated by extracellular signal-regulated kinases (ERK) 1/2 signaling pathways. These findings indicate that airway inflammation is controlled in a negative feedback manner involving MyD88s^22^. Negative feedback regulators of inflammation have been shown to be induced by inflammatory stimuli and play an essential role in maintaining immune homeostasis^23^. An alternative strategy for developing anti-inflammatory therapeutics is to up-regulate expression of the negative regulators of inflammation. Furthermore, MyD88s may prove to be an effective therapeutic target for regulating inflammation associated with airway disease.

Phosphodiesterase (PDE) inhibitors are widely studied as potential therapeutics for airway inflammation^24^. One such inhibitor, resveratrol, has been studied for a wide array of diseases. Resveratrol is a naturally occurring polyphenol found in plants such as grapes, berries, and nuts. Studies have shown that it inhibits the inflammatory response to LPS. All models used to study resveratrol have shown that its anti-inflammatory properties are equal to or superior to glucocorticoids^25^,^26^,^27^. This natural product directly targets PDE 1, 3 and 4 isoforms and has been shown to inhibit ERK and c-Jun N-terminal kinase (JNK) phosphorylation^28^,^29^,^30^. Since we have previously shown that ERK is a negative regulator for MyD88s, we postulated that resveratrol may be an effective therapeutic agent for the treatment of NTHi-induced airway inflammation by enhancing MyD88s expression via modulation of ERK.

Here we demonstrate for the first time that resveratrol decreases NTHi-induced lung inflammation by enhancing expression of MyD88s, a negative regulator of the NF-κB-dependent inflammatory response *in vitro* and in the lung of mice. This is accomplished by suppression of ERK-mediated down-regulation of MyD88s. Furthermore, resveratrol-mediated suppression of ERK phosphorylation occurs as a result of increased MAP kinase phosphatase-1 (MKP-1) expression via a cAMP-PKA-dependent mechanism. Our study suggests that MyD88s is an effective therapeutic target and unveils a novel mechanism by which resveratrol suppresses lung inflammation via up-regulation of the negative regulator of inflammation MyD88s.

## Results

### Resveratrol suppresses NTHi-induced inflammation in airway epithelial cells *in vitro* and in the lung of mice

**Figure 1:**
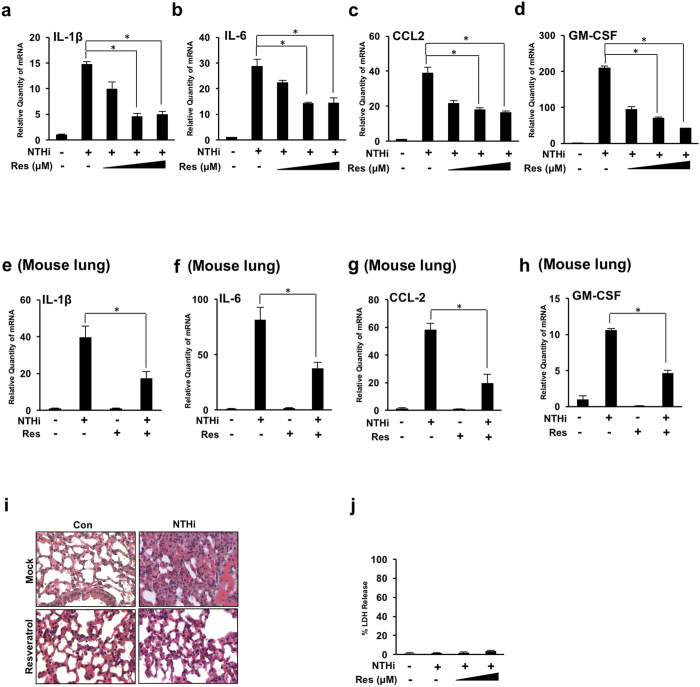
Resveratrol suppresses NTHi-induced lung inflammation in airway epithelial cells and in the lung of mice. (**a–d**) Relative quantities of human IL-1β (**a**) human IL-6 (**b**) human CCL-2 (**c**) and human GM-CSF (**d**) mRNA were measured by real-time QPCR analysis in human bronchial epithelial BEAS-2B cells. Cells were treated with 10 μM, 25 μM or 50 μM of resveratrol followed by NTHi stimulation. (**e–i**) C57BL/6 mice were intraperitoneally inoculated with resveratrol 1 hour before intratracheal inoculation with NTHi or saline for the control. Relative quantities of murine IL-1β (**e**), murine IL-6 (**f**), murine CCL-2 (**g**) and murine GM-CSF (**h**) mRNA were measured by real-time QPCR analysis of RNA extracted from lung tissue. (**i**) Lung tissue was harvested for histological analysis (H&E stain, 400x). (**j**) Cells were treated with resveratrol and NTHi for 24 hours. Cytotoxicity was determined by measuring lactate dehydrogenase release from BEAS-2B cells. Data are mean ± SD (*n* = 3). *p < *0.05*. Statistical analysis was performed using Student’s *t-*test. n.s., nonsignificant. Data are representative of three or more independent experiments.

Previous studies have shown that resveratrol reduces expression of proinflammatory cytokines^29^. Human bronchial smooth muscle cells treated with resveratrol showed a decrease in pro-inflammatory cytokine release in response to lipoteichoic acid stimulation^26^. Therefore, we first sought to determine if resveratrol can reduce NTHi-induced inflammation in bronchial epithelial (BEAS-2B) cells. As shown in (Fig. 1a–d), resveratrol significantly suppresses NTHi-induced IL-1β, IL-6, CCL-2 and GM-CSF mRNA expression in a dose-dependent manner. Resveratrol also has been shown to reduce lung tissue inflammation in a murine model of allergic airway disease^25^. Similar to the *in vitro* findings, resveratrol suppresses NTHi-induced IL-1β, IL-6, CCL-2 and GM-CSF mRNA expression in the lung of mice (Fig. 1e–h). A histological analysis was performed from lung tissue to verify the anti-inflammatory effects of resveratrol. Resveratrol reduces NTHi-induced leukocyte infiltration (Fig. 1i). To ensure these findings are not a result of cytotoxicity, lactate dehydrogenase (LDH) release was measured and no significant cytotoxicity was observed (Fig. 1j).

### Resveratrol suppresses NTHi-induced inflammation by up-regulating MyD88 short expression

**Figure 2:**
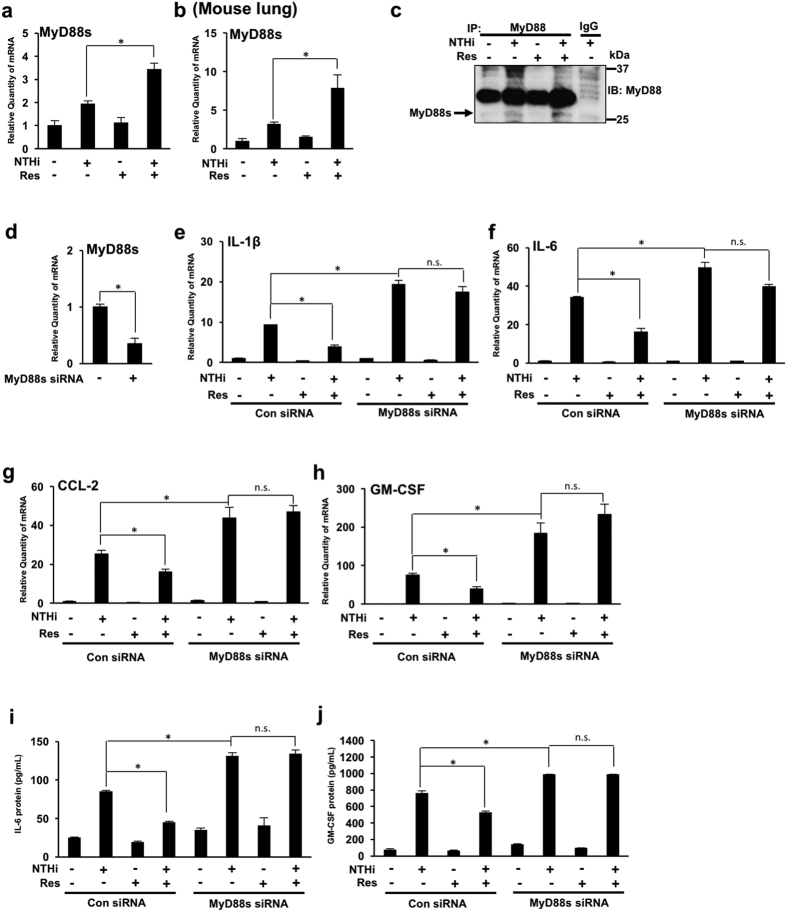
Resveratrol suppresses NTHi-induced inflammation by up-regulating MyD88 short expression. (**a**) BEAS-2B cells were treated with resveratrol 1 hour prior to NTHi stimulation. Relative quantities of human MyD88s mRNA were measured by real-time QPCR analysis. (**b)** C57BL/6 mice were intraperitoneally inoculated with resveratrol 1 hour before intratracheal inoculation with NTHi or saline for the control. Relative quantities of mouse MyD88s mRNA were measured by real-time QPCR analysis. (**c**) Cells were treated with resveratrol 1 hour prior to NTHi stimulation. MyD88 protein was immunoprecipitated from cell lysates and western blot analysis was performed to detect MyD88s expression as indicated by the arrow (27 kDa). (**d–j**) Cells were transfected with control siRNA or MyD88s siRNA followed by resveratrol treatment and NTHi stimulation. Relative quantities of human MyD88s (**d**) human IL-1β (**e**), human IL-6 (**f**), human CCL-2 (**g**) and human GM-CSF (**h**) mRNA were measured by real-time QPCR analysis. IL-6 (**i**) and GM-CSF (**j**) protein were measured by ELISA. Data are mean ± SD (*n *= 3). *p < *0.05*. Statistical analysis was performed using Student’s *t-*test. n.s., nonsignificant. Data are representative of three or more independent experiments.

We previously showed that NTHi induces expression of MyD88s *in vitro* and in the lung of mice. We also showed that MyD88s is a negative regulator of inflammation in airway epithelial cells^22^. An alternative strategy for the development of anti-inflammatory therapeutics is to up-regulate the negative regulators of inflammation. We thus hypothesized that the anti-inflammatory effects of resveratrol are a result of up-regulation of MyD88s. Interestingly, we found that resveratrol significantly enhances NTHi-induced MyD88s mRNA expression in BEAS-2B cells and in the lung of mice (Fig. 2a,b). Immunoprecipitation and western blot analysis were performed to confirm that resveratrol increases MyD88s protein expression *in vitro* (Fig. 2c). We then used MyD88s siRNA to further confirm that resveratrol decreases NTHi-induced proinflammatory cytokine expression by increasing MyD88s expression (Fig. 2d–j). Resveratrol no longer significantly reduces NTHi-induced IL-1β, IL-6, CCL-2 and GM-CSF mRNA expression in cells transfected with MyD88s siRNA (Fig. 2e–h). Furthermore, ELISA shows that resveratrol decreases NTHi-induced IL-6 and GM-CSF protein expression in BEAS-2B cells. However, resveratrol no longer significantly decreases NTHi-induced IL-6 and GM-CSF protein expression in cells transfected with MyD88s siRNA (Fig. 2i,j). Together these data suggest that resveratrol suppresses NTHi-induced lung inflammation by up-regulating MyD88s expression.

### Resveratrol up-regulates NTHi-induced MyD88 short via suppressing ERK1/2 phosphorylation

**Figure 3:**
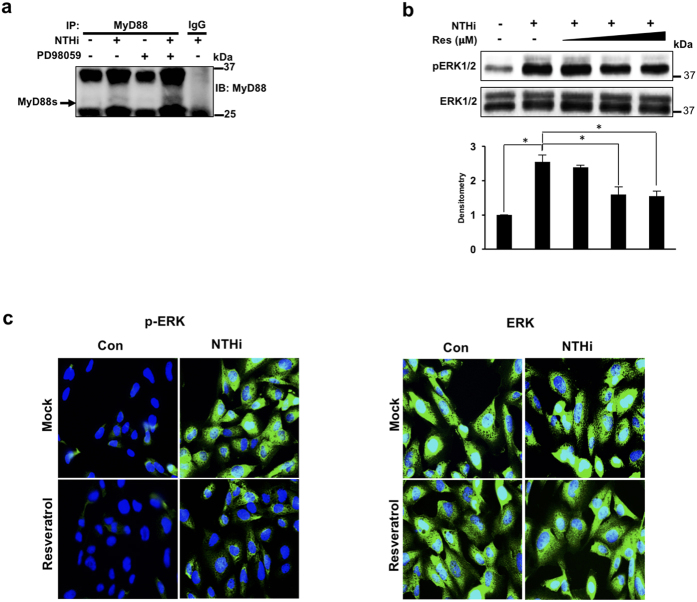
Resveratrol up-regulates NTHi-induced MyD88 short expression via suppressing ERK1/2 phosphorylation. (**a**) BEAS-2B cells were treated with PD98059 1 hour prior to NTHi stimulation. MyD88 protein was immunoprecipitated from cell lysates and western blot analysis was performed to detect MyD88s expression as indicated by the arrow (27 kDa). (**b**) Western blot analysis of phospho-ERK1/2 and total ERK1/2. Cells were treated with 10 μM, 25 μM or 50 μM of resveratrol followed by NTHi stimulation for 30 minutes. The fold change of phosphorylated ERK was quantified by densitometry and normalized to control. (**c**) Cells were treated with 25 μM of resveratrol followed by NTHi stimulation for 30 minutes. Phosphorylated ERK1/2 and total ERK1/2 were detected by immunofluorescence. Displayed immunoblots are cropped images from full-length blots, present in Supplementary Figure S1. Data are representative of at least three independent experiments.

We have previously shown that MyD88s is negatively regulated by the ERK1/2 signaling pathway. Chemically inhibiting ERK1/2 and ERK1/2 knockdown in BEAS-2B cells enhanced NTHi-induced MyD88s mRNA expression^22^. To confirm the regulatory role of ERK on MyD88s protein expression, BEAS-2B cells were treated with the ERK inhibitor PD98059 followed by NTHi stimulation. Immunoprecipitation and western blot analysis shows that ERK inhibition increases NTHi-induced MyD88s protein expression (Fig. 3a). Studies have shown that resveratrol suppresses tumor growth factor (TGF)-β-induced ERK1/2 phosphorylation^27^. Therefore, we determined whether resveratrol suppresses NTHi-induced ERK1/2 phosphorylation in BEAS-2B cells. We found that resveratrol decreases NTHi-induced ERK1/2 phosphorylation in a dose-dependent manner by western blot analysis (Fig. 3b). Immunofluorescence staining was then used to confirm these effects. Consistent with western blot analysis, immunofluorescence staining shows that resveratrol significantly reduces NTHi-induced ERK1/2 phosphorylation (Fig. 3c). Together these data suggest that resveratrol suppresses ERK-dependent down-regulation of MyD88s to reduce lung inflammation.

### Resveratrol enhances MyD88 short expression via up-regulating MKP-1

**Figure 4:**
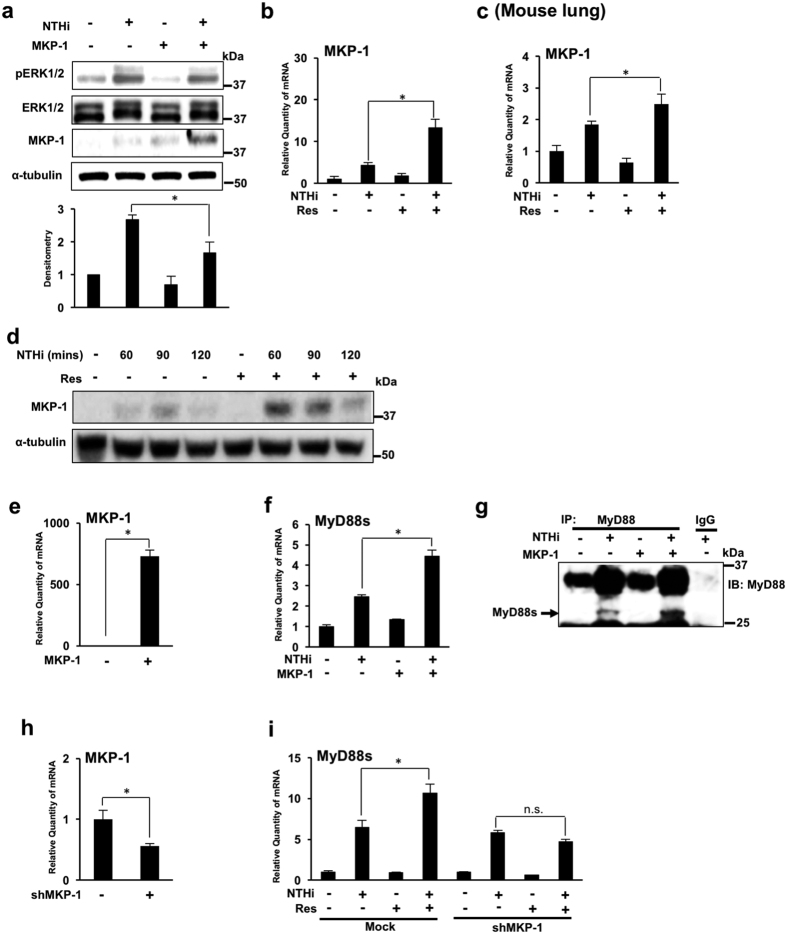
Resveratrol enhances MyD88 short expression via up-regulating MKP-1. (**a**) BEAS-2B cells were transfected with an MKP-1 overexpression vector and stimulated with NTHi for 30 minutes. Phosphorylated ERK1/2, total ERK1/2, MKP-1 and α-tubulin proteins were visualized via western blot analysis. The fold change of phosphorylated ERK was quantified by densitometry and normalized to control. (**b**) Relative quantities of human MKP-1 mRNA from BEAS-2B cells, treated with resveratrol followed by NTHi stimulation, were measured by real-time QPCR analysis. (**c**) Relative quantities of murine MKP-1 mRNA from the lung tissue of C57BL/6 mice, inoculated with resveratrol 1 hour prior to NTHi stimulation, were measured by real-time QPCR analysis. (**d**)Western blot analysis of MKP-1 and α-tubulin in BEAS-2B cells treated with resveratrol and stimulated with NTHi for indicated times. (**e–g**) BEAS-2B cells were transfected with a MKP-1 expression plasmid and stimulated with NTHi. Relative quantities of human MKP-1 (**e**) and human MyD88s (**f**) mRNA were measured by real-time QPCR analysis. Immunoprecipitation and western blot analysis were used to detect MyD88s protein expression as indicated by the arrow (27 kDa) (**g**). (**h,i**) BEAS-2B were transfected with empty vector or shMKP-1. Relative quantities of human MKP-1 (**h**) and human MyD88s (**i**) mRNA were measured by real-time QPCR analysis. Data are mean ± SD (*n* = 3). *p < *0.05*. Statistical analysis was performed using Student’s *t-*test. n.s., nonsignificant. Displayed immunoblots are cropped images from full-length blots, present in Supplementary Figure S2. Data are representative of three or more independent experiments.

To further elucidate the molecular mechanisms by which resveratrol enhances NTHi-induced MyD88s, we investigated the role of MKP-1. MKP-1 is known to negatively regulate TLR-dependent immune responses by dephosphorylating the MAP kinase ERK^31^,^32^. An MKP-1 overexpression vector was used to determine that MKP-1 is a negative regulator of ERK1/2 phosphorylation in airway epithelial cells. As expected, MKP-1 overexpression reduces NTHi-induced ERK1/2 phosphorylation (Fig. 4a). Additionally, bacterial stimulation has been shown to induce MKP-1 mRNA expression in epithelial cells^33^. We thus postulated that resveratrol may mediate ERK1/2 phosphorylation by increasing NTHi-induced MKP-1 mRNA expression. As shown in Fig. 4b,c, NTHi induces MKP-1 mRNA expression, and interestingly, resveratrol significantly enhances MKP-1 mRNA expression in BEAS-2B cells and in the lung of mice. Western blot analysis shows that resveratrol increases MKP-1 protein expression *in vitro* (Fig. 4d). We next examined the direct role of MKP-1 on MyD88s expression by overexpressing MKP-1 (Fig. 4e,f). MKP-1 overexpression significantly enhances NTHi-induced MyD88s mRNA expression (Fig. 4f). Immunoprecipitation and western blot analysis were performed to show that MKP-1 overexpression increases NTHi-induced MyD88s protein expression (Fig. 4g) Finally, shMKP-1 was used to determine if resveratrol enhances MyD88s via increasing MKP-1(Fig. 4h,i). Resveratrol no longer enhances NTHi-induced MyD88s mRNA in cells transfected with shMKP-1 (Fig. 4i). Together these data demonstrate that resveratrol suppresses ERK-dependent down-regulation of NTHi-induced MyD88s expression via up-regulating MKP-1 expression.

### NTHi-induced MyD88 short expression is mediated by MKP-1 up-regulation via a cAMP-PKA-dependent mechanism

**Figure 5:**
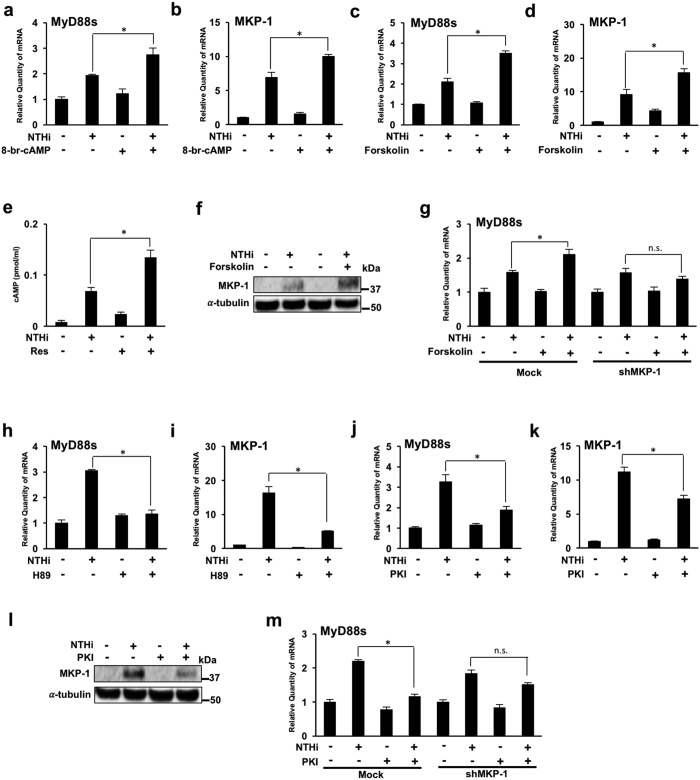
NTHi-induced MyD88 short expression is mediated by MKP-1 up-regulation via a cAMP-PKA-dependent mechanism. (**a,b**) BEAS-2B cells were treated with 8-bromo-cAMP 1 hour prior to NTHi stimulation. Relative quantities of human MyD88s (**a**) and human MKP-1 (**b**) mRNA were measured by real-time QPCR analysis. (**c,d**) Cells were treated with forskolin 1 hour prior to NTHi stimulation. Relative quantities of human MyD88s (**c**) and human MKP-1 (**d**) mRNA were measured by real-time QPCR analysis. (**e**) Cells were treated with resveratrol followed by NTHi and cell lysate was used to determine cAMP levels. (**f**) Western blot analysis of MKP-1 expression. Cells were treated with forskolin for 1 hour and stimulated with NTHi for 90 minutes. (**g**) Cells were transfected with shMKP-1 or empty vector and then treated with forskolin followed by NTHi stimulation. Relative quantities of human MyD88s mRNA were measured by real-time QPCR analysis. (**h,i**) Cells were treated with H89 1 hour prior to NTHi stimulation. Relative quantities of human MyD88s (**h**) and human MKP-1 (**i**) mRNA were measured by real-time QPCR analysis. (**j,k**) Cells were treated with PKI 1 hour prior to NTHi stimulation. Relative quantities of human MyD88s (**j**) and human MKP-1 (**k**) mRNA were measured by real-time QPCR analysis. (**l**) Western blot analysis of MKP-1 protein expression. Cells were treated with PKI for 1 hour followed by NTHI stimulation for 90 mins. (**m**) Cells were transfected with shMKP-1 or empty vector and then treated with PKI followed by NTHi stimulation. Relative quantities of human MyD88s mRNA were measured by real-time QPCR analysis. Data are mean ± SD (*n* = 3). *p < *0.05*. Statistical analysis was performed using Student’s *t-*test. Displayed immunoblots are cropped images from full-length blots, present in Supplementary Figure S3. Data are representative of three or more independent experiments.

Resveratrol is known to directly inhibit PDE1, 3 and 4 25,26. PDEs catalyze and degrade cAMP, an important second messenger with a critical role in regulating immune responses. PDE inhibitors therefore increase intracellular cAMP levels^34^. We therefore tested if cAMP plays a role in resveratrol-mediated MyD88s and MKP-1 enhancement using 8-bromo-cAMP, a cell-permeable cAMP analog resistant to PDE degradation. 8-bromo-cAMP treatment increases NTHi-induced MyD88s and MKP-1 mRNA expression (Fig. 5a,b). Forskolin, a cell permeable diterpenoid used to raise intracellular levels of cAMP, was then used to determine the role of cAMP in resveratrol-mediated MyD88s and MKP-1 expression. Forskolin treatment significantly increases NTHi-induced MyD88s and MKP-1 mRNA expression (Fig. 5c,d). To confirm the role of cAMP in resveratrol- mediated MyD88 and MKP-1 expression, we measured the effects of resveratrol on cAMP levels. Resveratrol significantly increases cAMP levels in BEAS-2B cells (Fig. 5e). Western blot analysis was performed to verify the effects of forskolin on NTHi-induced protein expression. As expected, forskolin increases NTHi-induced MKP-1 protein expression (Fig. 5f). Furthermore, forskolin no longer increases MyD88s mRNA expression in cells transfected with shMKP-1 confirming that cAMP acts upstream of MKP-1 (Fig. 5g). Together, these data suggest that cAMP is required for MKP-1 dependent up-regulation of NTHi-induced MyD88s.

A major downstream signaling effector of cAMP is protein kinase A (PKA) and the cAMP-PKA signaling pathway has been shown to regulate MKP-1 expression^32^,^33^. Therefore, we tested if PKA is involved in resveratrol-mediated MyD88s and MKP-1 expression. Cells treated with PKA inhibitor H89, followed by NTHi stimulation show a significant reduction of MyD88s and MKP-1 mRNA expression (Fig. 5h,i). PKI, a PKA-specific inhibitor, was further used to confirm PKA involvement. PKI treatment significantly decreases MyD88s and MKP-1 mRNA expression (Fig. 5j,k). Western blot analysis was then performed to confirm the effects of PKI on NTHi-induced protein expression. As expected, PKI treatment increases NTHi-induced MKP-1 protein expression (Fig. 5l). Furthermore, PKI no longer increases MyD88s mRNA expression in cells transfected with shMKP-1, confirming that PKA acts upstream of MKP-1 (Fig. 5m). Collectively, these data suggest that resveratrol mediates MKP-1-dependent up-regulation of MyD88s expression via a cAMP-PKA-dependent mechanism.

### Treatment with resveratrol post NTHi infection enhances MyD88 short expression and decreases expression of proinflammatory mediators *in vitro* and in the lung of mice

**Figure 6:**
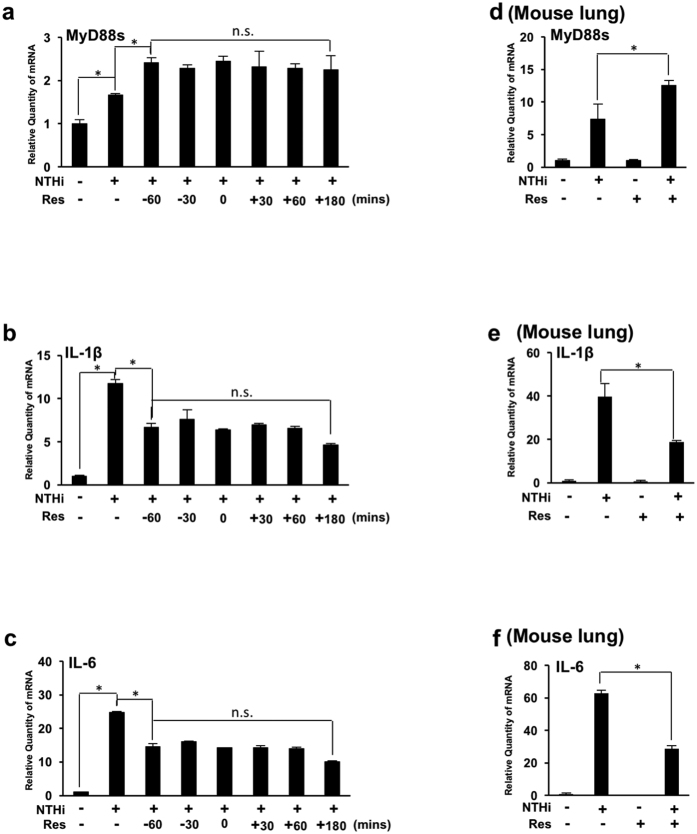
Treatment with resveratrol post NTHi infection enhances MyD88 short expression and decreases expression of proinflammatory mediators *in vitro* and in the lung of mice. (**a**–**c**) BEAS-2B cells were treated with resveratrol at indicated times relative to NTHi stimulation. Relative quantities of human MyD88s (**a**) human IL-1β (**b**) and human IL-6 (**c**) mRNA were measured via real-time QPCR analysis. (**d**–**f**) Mice were inoculated with resveratrol 3 hours post NTHi inoculation. Relative quantities of murine MyD88s (**d**), murine IL-1β (**e**) and murine IL-6 (**f**) mRNA from lung tissue were measured by real-time QPCR analysis. Data are mean ± SD (*n* = 3). *p < *0.05*. Statistical analysis was performed using Student’s *t-*test. n.s., nonsignificant. Data are representative of three or more independent experiments.

We have demonstrated that pre-administration of resveratrol significantly decreases NTHi-induced lung inflammation via up-regulation of MyD88s. Furthermore, we have previously shown that treatment with other PDE inhibitors post infection has therapeutic potential^32^. We thus sought to validate the physiological relevance of resveratrol to the clinical situation. BEAS-2B cells were treated with resveratrol at various times pre- and post-NTHi stimulation. Our data show that resveratrol increases NTHi-induced MyD88s mRNA expression and decreases NTHi-induced IL-1β and IL-6 mRNA expression (Fig. 6a–f). Furthermore, there is no significant change in MyD88s, IL-1β and IL-6 mRNA expression among cells treated with resveratrol before and after NTHi stimulation (Fig. 6a–c). Mice were also treated with resveratrol 3 hours post NTHi stimulation. Consistent with the *in vitro* data, treatment with resveratrol post NTHi stimulation increases MyD88s and decreased IL-1β and IL-6 mRNA levels in lung tissue of mice (Fig. 6d–f). Together these data confirm the physiological relevance for the clinical use of resveratrol for the treatment of NTHi-induced lung inflammation.

## Discussion

Asthma and COPD affect more than one-half billion people globally resulting in billions of dollars in healthcare costs, and there are no cures^1^,^2^,^3^,^4^,^5^. These diseases are characterized by excessive inflammation that is often exacerbated by bacterial respiratory infections, such as NTHi^6^,^7^. The increasing numbers of antibiotic-resistant NTHi strains, tolerance issues and adverse side effects of currently available therapies, together with the increasing prevalence of airway diseases requires the development of novel, non-antibiotic therapeutics^5^,^10^,^11^. Resveratrol is being widely studied for its therapeutic potential for a vast array of diseases^28^,^29^,^30^. However, the molecular mechanisms by which resveratrol exerts its anti-inflammatory effects are still largely unknown. We have previously shown that MyD88s is a negative regulator of NTHi-induced inflammation in airway epithelial cells and that MyD88s is negatively regulated by ERK1/2 signaling pathways in a negative feedback manner suggesting that MyD88s is essential for maintaining immune homeostasis^22^.

**Figure 7:**
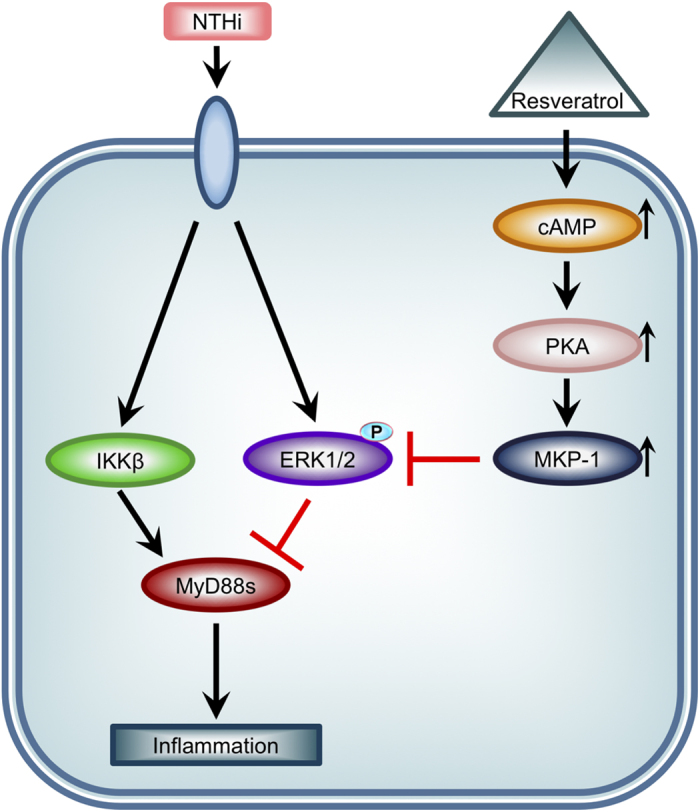
Resveratrol alleviates NTHi-induced inflammation by counteracting ERK-dependent down-regulation of MyD88s via a cAMP-PKA-MKP-1 pathway. The anti-inflammatory effects of resveratrol are mediated by a cAMP-PKA-MKP-1 pathway that suppresses of ERK-dependent down-regulation of NTHi-induced MyD88s in airway epithelial cells.

In this study we show that resveratrol decreases NTHi-induced expression of pro-inflammatory mediators in airway epithelial cells and in the lung of mice. We demonstrate for the first time that resveratrol suppresses NTHi-induced inflammation by up-regulating MyD88s, a negative regulator of inflammation, via inhibition of ERK1/2 phosphorylation. Moreover, resveratrol mediates ERK-dependent down-regulation of MyD88s via up-regulation of MKP-1, a critical negative regulator of ERK. Furthermore, resveratrol enhances MKP-1 expression via a cAMP-PKA-dependent mechanism. Finally, we show that treatment with resveratrol post NTHi stimulation supports the clinical relevance of the use of resveratrol for the treatment of NTHi-induced lung inflammation. Together these data reveal a novel mechanism of the anti-inflammatory effects of resveratrol by which resveratrol alleviates NTHi-induced inflammation partially by counteracting ERK-dependent down-regulation of MyD88s via a cAMP-PKA-MKP-1 pathway (Fig. 7).

Many ongoing studies focus on the therapeutic potential of resveratrol with promising anti-inflammatory and airway remodeling results in respiratory disease models^25^,^26^,^27^. Concurrently, our findings suggest that resveratrol usage for acute lung inflammation has clinical significance. Human clinical trials have been performed with patients for cancer, cardiovascular disease and metabolic diseases. However, the results of these studies are not as promising as *in vitro* and *in vivo* studies with many failing to demonstrate any anti-inflammatory effects with resveratrol. These discrepancies among clinical trials have been attributed to study populations or design with a key factor being the limited bioavailability of resveratrol^35^,^36^. Metabolism in humans is rapid with conversion to metabolites within 30 minutes^28^. Therefore, identifying the molecular mechanisms of resveratrol’s promising anti-inflammatory effects may lead to the development of better therapeutics with improved clinical trial outcomes. Furthermore, MyD88s may be a critical therapeutic target with significant therapeutic potential for suppressing inflammation associated with chronic airway disease.

## Materials and Methods

### Cell Culture

Human bronchial epithelial cells BEAS-2B cells (ATCC) were maintained in RPMI 1640 medium (Gibco) supplemented with 10% (v/v) heat-inactivated FBS (Sigma-Aldrich) and 100 units/mL penicillin and 0.1 mg/mL streptomycin. Cells were cultured at 37 °C in a humidified atmosphere of 5% CO_2_ with a passage number no greater than 25 23,35. Cells were seeded in 12 well plates for all experiments.

### Bacteria Strain and Culture Conditions

A clinical isolate of NTHi strain 12 was grown on chocolate agar plates at 37 °C in 5% CO_2_ overnight, harvested and incubated overnight in brain heart infusion (BHI) broth supplemented with 3.5 μg/ml NAD and hemin. Bacteria were subcultured in fresh BHI broth to log phase growth, as measured by optical density, pelleted washed, and resuspended in DMEM for *in vitro* experiments or isotonic saline for *in vivo* experiments. BEAS-2B cells were stimulated with NTHi at a multiplicity of infection (MOI) of 50 unless otherwise specified for 6 hours or as indicated.

### Reagents and Antibodies

Resveratrol was purchased from Calbiochem. PD98059, 8-bromo-cAMP, and forskolin were purchased from Enzo Life Sciences. PKI and H89 were purchased from EMD Millipore. PKI and 8-br-cAMP were reconstituted in water and diluted with RPMI 1640 media. All other inhibitors were reconstituted in dimethyl sulfoxide (DMSO) and diluted with RPMI 1640 media to a final concentration of 0.1% DMSO. 0.1% DMSO in RPMI 1640 media was used as a control. Forty-eight hours after seeding, cells were treated with 25 μM of resveratrol or as indicated, 10 μM of PD98059, 50 μM of 8-bromo-cAMP, 10 μM of forskolin, 50 μM of PKI, or 20 μM of H89 in a final volume of 500 μL per well of 12 well plates one hour before stimulation with NTHi or as indicated. Antibody against MyD88 (ab133739) was purchased from Abcam. Antibodies against phospho-ERK1/2 (# 9101), total ERK1/2 (# 9102) and HRP-conjugated rabbit (# 7074) or mouse IgG (# 7076) were purchased from Cell Signaling Technology. Antibodies against MKP-1(# 370), α-tubulin (# 69969), normal rabbit IgG (#2027) and FITC-conjugated goat anti-mouse IgG (# 2010) were purchased from Santa Cruz Biotechnology.

### RNA Isolation and Real-time Quantitative RT-PCR (Q-PCR)

Q-PCR analysis of human and mouse MyD88s, IL-1β, IL-6, CCL-2, GM-CSF, and MKP-1 was conducted as follows. Following transfection and/or treatment with resveratrol or other inhibitors and/or NTHi stimulation, total RNA was isolated from BEAS-2B cells with TRIzol reagent (Invitrogen) according to the manufacturer’s instructions. Reverse transcription was performed using 1 μg of RNA and TaqMan reverse transcription reagents; 10x RT buffer, MgCl_2_, dNTPs, random hexamers, oligo (dT) primers, RNase inhibitor and reverse transcriptase (Applied Biosystems). The reaction was performed for 10 minutes at 25 °C followed by 60 minutes at 42 °C. SYBR Green Universal Master Mix (Applied Biosystems) was used for the PCR amplification. In brief, reactions were performed in triplicate containing 2 x universal master mix, 1 μL of template cDNA, 500 nM primers in a final volume of 25 μL, and they were analyzed in a 96-well optical reaction plate (Applied Biosystems). An ABI 7500 sequence detector with accompanying software (Applied Biosystems) was used for amplification and quantification. The comparative threshold cycle (Ct) method was used to obtain relative quantities of mRNAs that were normalized using human cyclophilin A or mouse glyceraldehyde-3-phosphate dehydrogenase (GAPDH) as an endogenous control. The primer sequences for human cyclophilin A, IL-6, IL-1β, and MyD88s as well as mouse GAPDH, IL-6 and IL-1β and MyD88s were described previously^22^,^23^,^37^,^38^. The primer sequences for human and mouse CCL-2, GM-CSF and MKP-1 are as follows: human CCL-2 forward primer, 5′-TCGCTCAGCCAGATGCAATC- 3′; human CCL-2 reverse primer, 5′-GACACTTGCTGCTGGTGATTC-3′; human GM-CSF forward primer, 5′-AACAGTAGAAGTCATCTCAGAAATGTTTG-3′; human GM-CSF reverse primer, 5′-GCTGGCCATCATGGTCAAG-3′; human MKP-1 forward primer, 5′-GCTGTGCAGCAAACAGTCGA-3′; human MKP-1 reverse primer, 5′-GCCACCCTGATCGTAG-3′; mouse CCL-2 forward primer, 5′-GAGTAGGCTGGAGAGCTACAAG-3′; mouse CCL-2 reverse primer, 5′-TGAGCTTGGTGACAAAAACTACAG-3′; mouse GM-CSF forward primer, 5′-ATGCCTGTCACGTTGAATGAAG-3′; mouse GM-CSF reverse primer, 5′-GCGGGTCTGCACACATGTTA-3′; mouse MKP-1 forward primer, 5′-GCTGTGCAGCAAACAGTCGA-3′; mouse MKP-1 reverse primer, 5′-CGATTAGTCCTCATAAGGTA-3′.

### Enzyme-linked immunosorbent assay (ELISA) and cAMP enzyme immunoassay

BEAS-2B cells were treated with resveratrol 1 hour prior to NTHi stimulation. Culture supernatants were collected 12 hours after NTHi stimulation and centrifuged to remove cell debris prior to the assay. Human IL-6 and GM-CSF protein were measured with LEGEND MAX Human IL-6 and Human GM-CSF ELISA kits (Biolegend, Inc.) according to the manufacturer’s instructions. cAMP levels were quantitatively measured using the Cyclic AMP Enzyme Immunoassay kit (Alfa Aesar) according to the manufacturer’s instructions.

### Cytotoxicity Assay

The CytoTox 96^®^ non-radioactive cytotoxicity assay (Promega) was performed according to the manufacturer’s instructions. Twenty-four hours after stimulation with 25 μM or 50 μM of resveratrol and NTHi, the supernatant was collected and cells were lysed. The supernatant was diluted 1:2 and lysate was diluted 1:10 with RPMI in a final volume of 50 μL in a 96 well assay plate in triplicate. 50 μL of RPMI was added in triplicate to correct for background. Each well was incubated with 50 μL of a substrate for 30 minutes at room temperature protected from light, and 50 μL of stop solution was added to each well. The absorbance was read at 490 nm, and the background values were subtracted from the sample readings. The percent LDH release was calculated as the absorbance of the supernatant divided by absorbance of the supernatant plus the absorbance of the lysate (endogenous LDH release).

### RNA-mediated Interference

siRNA for MyD88s, 5′-CCCAGCATTGGGCATATGCCT-3′ and ON-TARGETplus non-targeting control pool were purchased from Dharmacon. At 80% confluence, cells were transfected with 20 nM MyD88s siRNA and 1 μL of DharmaFECT (Dharmacon) in 1000 μL of media per well according to the manufacturer’s instructions. shMKP-1 was generated as previously described^39^. MKP-1 knockdown was performed using 0.8 μg DNA and 1 μL Lipofectamine 3000 (Invitrogen) following the manufacturer’s instructions. Forty-eight hours after transfection, cells were treated with resveratrol and stimulated with NTHi. The cell lysate was used for mRNA analysis.

### Western Blot Analysis, Immunoprecipitation and Immunofluorescence

Western blot analyses were performed as previously described^33^. Following treatment with resveratrol and NTHi stimulation, cells were lysed, incubated on ice for 30 minutes and centrifuged at 12,000 × *g* for 15 minutes. Supernatants were separated on a 10% SDS-PAGE gel, transferred to polyvinylidene fluoride membrane. The membranes were blocked with a solution of Tris-buffered saline (TBS) containing 0.1% Tween 20 (TBS-T) and 5% nonfat dry milk, then incubated with primary antibodies against phospho-ERK1/2 or total ERK1/2 at a 1:2000 dilution or MyD88, MKP-1 or α-tubulin at a 1:1000 dilution in 5% BSA-TBS-T overnight. After three washes in TBS-T, the membrane was incubated with secondary HRP-conjugated rabbit or mouse IgG antibody at a 1:5000 dilution in 5% nonfat dry milk-TBS-T for 1 hour. Proteins were visualized using Amersham ECL Prime Western Blotting Detection Reagent (GE Healthcare Biosciences). Images were cropped for presentation. Full-size images are presented in Supplementary Figs 1–3. For immunoprecipitation, cell lysates were incubated with 4 μL of antibodies against MyD88 or normal IgG and conjugated to protein G plus agarose beads (Santa Cruz Biotechnology) overnight at 4 °C. Eluted protein was subjected to western blot analysis. For immunofluorescence, cells were cultured on four-chamber microscope slides. After treatment with resveratrol and NTHi, the cells were fixed in 4% paraformaldehyde solution and incubated with antibodies against phospho-ERK1/2 or total ERK for 1 hour. FITC-conjugated goat anti-rabbit IgG was used to detect primary antibody. Slides were viewed and photographed using a Zeiss Axiophot microscope.

### Plasmid and Transfection

The wild-type expression plasmid MKP-1 was previously described^40^. Cells were seeded in 12 well plates and at 80% confluence transfected with 0.8 μg using TransIT-LT-1 reagent (Mirus) according to the manufacturer’s instructions. Forty-eight hours after transfection, cells were stimulated with or without NTHi. The cell lysate was used for mRNA analysis or immunoprecipitation and western blot analysis.

### Mice and Animal Experiments

C57BL/6 mice were purchased from the Jackson Laboratory. Anaesthetized mice were intraperitoneally inoculated with 20 mg/kg resveratrol 1 hour before or 3 hours post intratracheal inoculation with NTHi at a concentration of 5 × 10^7^ CFU per mouse or saline as control. Mice were sacrificed by intraperitoneal injection with 100 mg/kg sodium pentobarbital 6 hours after bacterial inoculation. Lung tissue was harvested for total RNA extraction as previously described^41^. For histological analysis, harvested lung tissue was fixed with 10% buffered formaldehyde, embedded in paraffin and sectioned at 4-μM thickness. Sections were then stained with hematoxylin and eosin (H&E) to visualize the inflammatory response. Stained sections were visualized and images were recorded under light microscopy systems (Axiovert 40 CFL, Axiocam MRC, and Axiovision LE Image system; Carl Zeiss) as previously described^42^. All animal studies were carried out in accordance with the policies of, and with approval from, the Institutional Animal Care and Use Committee of Georgia State University.

### Statistical Analysis

All experiments were repeated in at least three independent experiments. Data are shown as the mean ± SD of *n* determinations. Statistical evaluation was done by unpaired Student’s *t*-test, and *p <* *0.05* was taken as a significant difference.

## Additional Information

**How to cite this article**: Andrews, C. S. *et al.* Resveratrol suppresses NTHi-induced inflammation via up-regulation of the negative regulator MyD88 short. *Sci. Rep.* **6**, 34445; doi: 10.1038/srep34445 (2016).

## Supplementary Material

Supplementary Information

## References

[b1] Olin J. T. & Wechsler M. E. Asthma: pathogenesis and novel drugs for treatment. BMJ. 349, g5517 (2014). 25420994 10.1136/bmj.g5517

[b2] Brusselle G. G., Joos G. F. & Bracke K. R. New insights into the immunology of chronic obstructive pulmonary disease. Lancet 378, 1015–1026 (2011). 21907865 10.1016/S0140-6736(11)60988-4

[b3] Myers T. R. & Tomasio L. Asthma: 2015 and beyond. Respir. Care 56, 1389–1407 (2011). 21944687 10.4187/respcare.01334

[b4] Hassett D. J., Borchers M. T. & Panos R. J. Chronic obstructive pulmonary disease (COPD): Evaluation from clinical, immunological and bacterial pathogenesis perspectives. J. Microbiol. 52, 211–226 (2014). 24585052 10.1007/s12275-014-4068-2

[b5] Ko F. W., Lim T. K., Hancox R. J. & Yang I. A. Year in review 2013: Chronic obstructive pulmonary disease, asthma and airway biology. Respirology 19, 438–447 (2014). 24708033 10.1111/resp.12252

[b6] Erle D. J. & Sheppard D. The cell biology of asthma. J. Cell Biol. 205, 621–631 (2014). 24914235 10.1083/jcb.201401050 PMC4050726

[b7] Barbu C., Iordache M. & Man M. G. Inflammation in COPD: pathogenesis, local and systemic effects. Rom. J. Morphol. Embryol. 52, 21–27 (2011). 21424028

[b8] Foxwell A. R., Kyd J. M. & Cripps A. W. Nontypeable Haemophilus influenzae: pathogenesis and prevention. Microbiol. Mol. Biol. Rev. 62, 294–308 (1998). 9618443 10.1128/mmbr.62.2.294-308.1998 PMC98916

[b9] Alikhan M. M. & Lee F. E. Understanding nontypeable Haemophilus influenzae and chronic obstructive pulmonary disease. Curr. Opin. Pulm. Med. 20, 159–164 (2014). 24441573 10.1097/MCP.0000000000000023

[b10] Schumacher S. K. *et al.* Prevalence and genetic diversity of nontypeable haemophilus influenzae in the respiratory tract of infants and primary caregivers. Pediatr. Infect. Dis. J. 31, 145–149 (2012). 22051860 10.1097/INF.0b013e31823aaeb3 PMC3261374

[b11] Ito M. *et al.* Clonal spread of beta-lactamase-producing amoxicillin-clavulanate-resistant (BLPACR) strains of non-typeable Haemophilus influenzae among young children attending a day care in Japan. Int. J. Pediatr. Otorhinolaryngol. 74, 901–906 (2010). 20846501 10.1016/j.ijporl.2010.05.008

[b12] Hirota J. A. & Knight D. A. Human airway epithelial cell innate immunity: relevance to asthma. Curr. Opin. Immunol. 24, 740–746 (2012). 23089231 10.1016/j.coi.2012.08.012

[b13] Sohal S. S., Ward C., Danial W., Wood-Baker R. & Walters E. H. Recent advances in understanding inflammation and remodeling in the airways in chronic obstructive pulmonary disease. Expert. Rev. Respir. Med. 7, 275–288 (2013). 23734649 10.1586/ers.13.26

[b14] Hallstrand T. S. *et al.* Airway epithelial regulation of pulmonary immune homeostasis and inflammation. Clin. Immunol. 151, 1–15 (2014). 24503171 10.1016/j.clim.2013.12.003

[b15] Proud D. & Leigh R. Epithelial cells and airway diseases. Immunol. Rev. 242, 186–204 (2011). 21682746 10.1111/j.1600-065X.2011.01033.x

[b16] Kawai T. & Akira S. The role of pattern-recognition receptors in innate immunity: update on Toll-like receptors. Nat. Immunol. 11, 373–384 (2010). 20404851 10.1038/ni.1863

[b17] Shuto T. *et al.* Activation of NF-kappa B by nontypeable Hemophilus influenzae is mediated by toll-like receptor 2-TAK1-dependent NIK-IKK alpha /beta-I kappa B alpha and MKK3/6-p38 MAP kinase signaling pathways in epithelial cells. Proc. Natl. Acad. Sci. USA. 98, 8774–8779 (2001). 11438700 10.1073/pnas.151236098 PMC37511

[b18] Mogensen T. H. Pathogen recognition and inflammatory signaling in innate immune defenses. Clin. Microbiol. Rev. 22, 240–273 (2009). 19366914 10.1128/CMR.00046-08 PMC2668232

[b19] Janssens S., Burns K., Tschopp J. & Beyaert R. Regulation of interleukin-1- and lipopolysaccharide-induced NF-kappaB activation by alternative splicing of MyD88. Curr. Biol. 12, 467–471 (2002). 11909531 10.1016/s0960-9822(02)00712-1

[b20] Burns K. *et al.* Inhibition of interleukin 1 receptor/Toll-like receptor signaling through the alternatively spliced, short form of MyD88 is due to its failure to recruit IRAK-4. J. Exp. Med. 197, 263–268 (2003). 12538665 10.1084/jem.20021790 PMC2193806

[b21] Janssens S., Burns K., Vercammen E., Tschopp J. & Beyaert R. MyD88s, a splice variant of MyD88, differentially modulates NF-kappaB- and AP-1-dependent gene expression. FEBS Lett. 548, 103–107 (2003). 12885415 10.1016/s0014-5793(03)00747-6

[b22] Andrews C. S. *et al.* Nontypeable *Haemophilus influenzae*-induced MyD88 short expression is regulated by positive IKKβ and CREB pathways and negative ERK1/2 pathway. PLoS One 10, e0144840 (2015). 26669856 10.1371/journal.pone.0144840 PMC4684398

[b23] Miyata M. *et al.* Glucocorticoids suppress inflammation via the upregulation of negative regulator IRAK-M. Nat. Commun. 6, 6062 (2015). 25585690 10.1038/ncomms7062 PMC4309435

[b24] Page C. P. & Spina D. Phosphodiesterase Inhibitors in the treatment of inflammatory diseases. Handb. Exp. Pharmacol. 204, 391–414 (2011). 21695650 10.1007/978-3-642-17969-3_17

[b25] Royce S. G. *et al.* Resveratrol has protective effects against airway remodeling and airway hyperreactivity in a murine model of allergic airway disease. Pathobiol. Aging. Age Relat. Dis. 1, 10.3402/PBA.v1i0.7134 (2011). PMC3417665 22953028

[b26] Knobloch J. *et al.* Resveratrol attenuates the release of inflammatory cytokines from human bronchial smooth muscle cells exposed to lipoteichoic acid in chronic obstructive pulmonary disease. Basic Clin. Pharmocol. Toxicol. 114, 202–209 (2014). 10.1111/bcpt.12129 23981542

[b27] Lee M. *et al.* Anti-inflammatory and anti-asthmatic effects of resveratrol, a polyphenolic stilbene, in a mouse model of allergic asthma. Int. Immunopharmacol. 9, 418–424 (2009). 19185061 10.1016/j.intimp.2009.01.005

[b28] Wood L. G., Wark P. A. & Garg M. L. Antioxidant and anti-inflammatory effects of resveratrol in airway disease. Antioxid. Redox. Signal. 13, 1535–1548 (2010). 20214495 10.1089/ars.2009.3064

[b29] Britton R. G., Kovoor C. & Brown K. Direct molecular targets of resveratrol: identifying key interactions to unlock complex mechanisms. Ann. N. Y. Acad. Sci. 1348, 124–133 (2015). 26099829 10.1111/nyas.12796

[b30] Kuroyanagi G. *et al.* Resveratrol suppresses TGF-β-induced VEGF synthesis in osteoblasts: Inhibition of the p44/p42 MAPK and SAPK/JNK pathways. Exp. Ther. Med. 9, 2303–2310 (2015). 26136978 10.3892/etm.2015.2389 PMC4473361

[b31] Kondoh K. & Nishida E. Regulation of MAP kinases by MAP kinase phosphotases. Biochim. Biophys. Acta. 1773, 1227–1237 (2007). 17208316 10.1016/j.bbamcr.2006.12.002

[b32] Liew F. Y., Xu D., Brint E. K. & O’Neill L. A. Negative regulation of toll-like receptor-mediated immune responses. Nat. Rev. Immunol. 5, 446–458 (2005). 15928677 10.1038/nri1630

[b33] Lee J. *et al.* Phosphodiesterase 4B mediates extracellular signal-regulated kinase-dependent up-regulation of mucin MUC5AC protein by Streptococcus pneumoniae by inhibiting cAMP-protein kinase A-dependent MKP-1 phosphatase pathway. J. Biol. Chem. 287, 22799–22811 (2012). 22610099 10.1074/jbc.M111.337378 PMC3391144

[b34] Susuki-Miyata S. *et al.* Cross-talk between PKA-Cβ and p65 mediates synergistic induction of PDE4B by roflumilast and NTHi. Proc. Natl. Acad. Sci. USA. 112, E1800–E1809 (2015). 25831493 10.1073/pnas.1418716112 PMC4394257

[b35] Park E. J. & Pezzuto J. M. The pharmacology of resveratrol in animals and humans. Biochim. Biophys. Acta. 1852, 1071–1113 (2015). 25652123 10.1016/j.bbadis.2015.01.014

[b36] Poulsen M. M. *et al.* Resveratrol and inflammation: Challenges in translating pre-clinical findings to improved patient outcomes. Biochim. Biophys. Acta. 1852, 1124–1136 (2015). 25583116 10.1016/j.bbadis.2014.12.024

[b37] Koga T. *et al.* Tumor suppressor cylindramatosis acts a negative regulator for Streptococcus pneumoniae-induced NFAT signaling. J. Biol. Chem. 283, 12546–12554 (2008). 18332137 10.1074/jbc.M710518200 PMC2335367

[b38] Jeon K. I. *et al.* Vinpocetine inhibits NF-kappaB-dependent inflammation via a IKK-dependent but PDE-independent mechanism. Proc. Natl. Acad. Sci. 107, 9795–9800 (2010). 20448200 10.1073/pnas.0914414107 PMC2906898

[b39] Wang W. Y. *et al.* CYLD negatively regulates nontypeable Haemophilus influenzae-induced IL-8 expression via phosphatase MKP-1-dependent inhibition of ERK. PLoS One 9, e112516 (2014). 25389768 10.1371/journal.pone.0112516 PMC4229244

[b40] Jono H. *et al.* Transforming growth factor-β-smad signaling pathway negatively regulates nontyypeable *Haemophilus influenzae*-induced MUC5AC mucin transcription via mitogen-activated protein kinase (MAPK) phosphatase-1-dependent inhibition of p38 MAPK. J. Biol. Chem. 278, 27811–27819 (2003). 12734193 10.1074/jbc.M301773200

[b41] Lim J. H. *et al.* Tumor suppressor CYLD acts as a negative regulator for non-typeable *Haemophilus influeza*-induced inflammation in the middle ear and lung of mice. PLoS One 10, e1032 (2007). 17925880 10.1371/journal.pone.0001032 PMC2001183

[b42] Lee B.-C., Miyata M., Lim J. H. & Li J. D. Deubiquitinase CYLD acts as a negative regulator for bacterium NTHi-induced inflammation by suppressing K63-linked ubiquitination of MyD88. Proc. Natl. Acad. Sci. 113, E165–E171 (2016). 26719415 10.1073/pnas.1518615113 PMC4720297

