# Supplementary material for: Resveratrol suppresses NTHi-induced inflammation via up-regulation of the negative regulator MyD88 short
Source: Sci Rep. 2016 Sep 28;6:34445. doi: 10.1038/srep34445 (PMC5039644; doi:10.1038/srep34445)
Supplement: Supplementary Information [file srep34445-s1.pdf]

Supplementary Information

Resveratrol suppresses NTHi-induced inflammation via up-regulation of the negative regulator MyD88 short

Carla S. Andrews, Shingo Matsuyama, Byung-Cheol Lee, and Jian-Dong Li

Fig. 3b

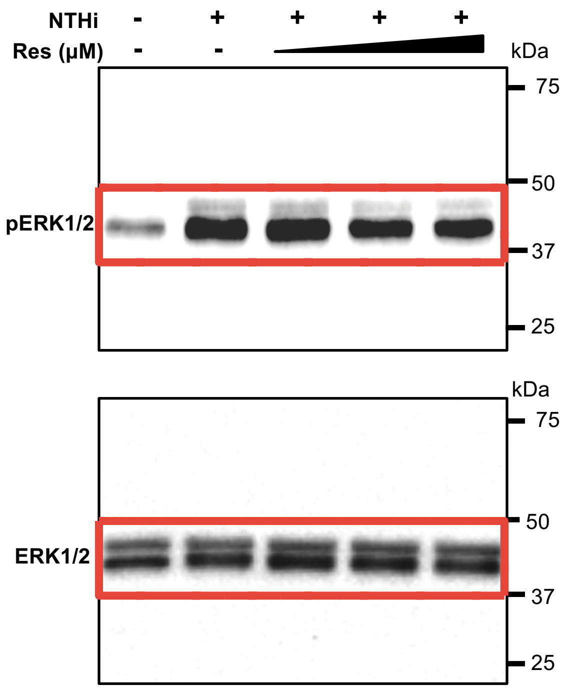

Supplementary Figure S1. Uncropped immunoblot images with molecular weight markers shown in Fig. 3

Fig. 4a

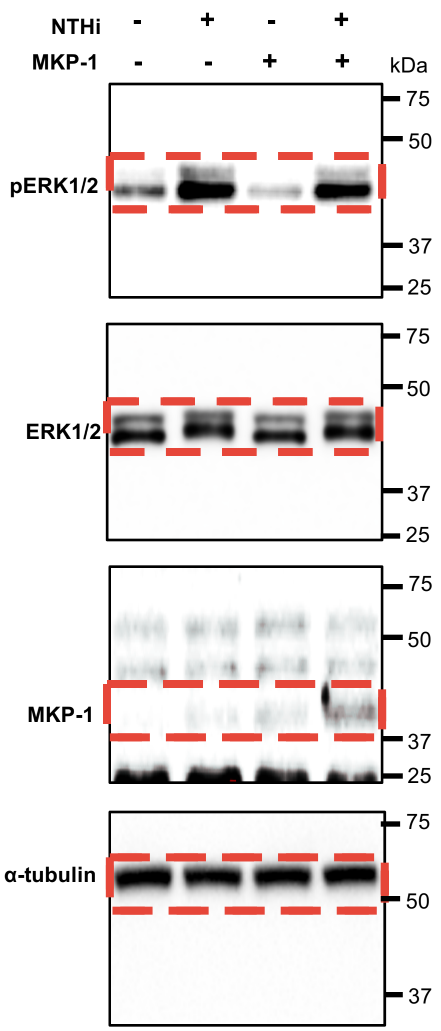

Fig. 4d

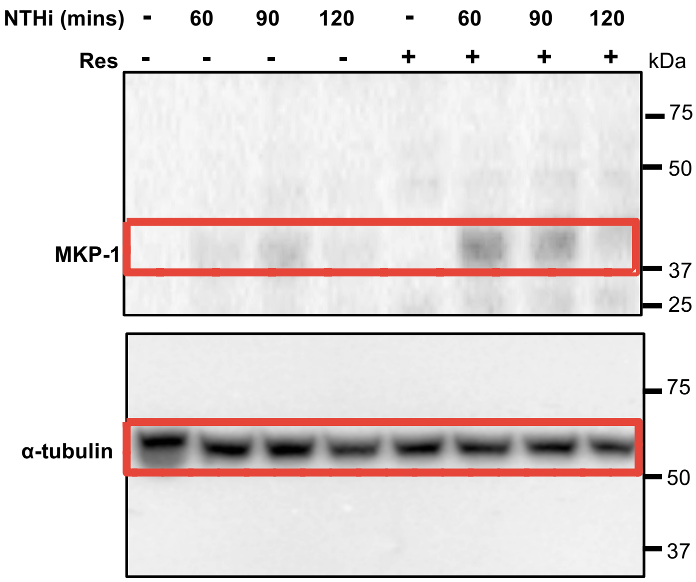

Supplementary Figure S2. Uncropped immunoblot images with molecular weight markers shown in Fig. 4

Fig. 5f

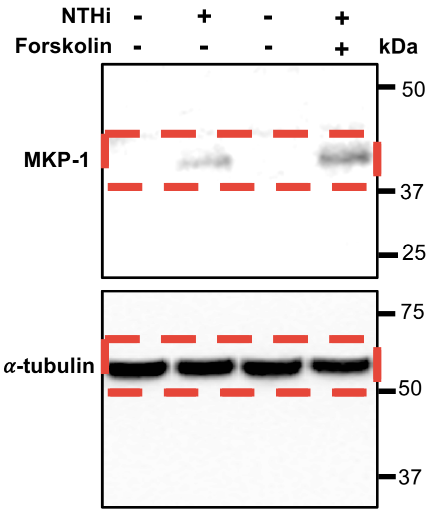

Fig. 5l

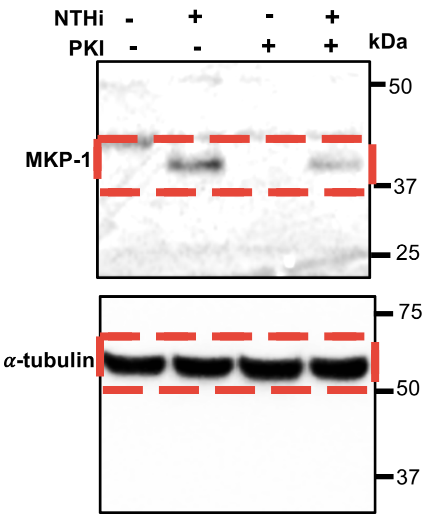

Supplementary Figure S3. Uncropped immunoblot images with molecular weight markers shown in Fig. 5
